# LM-SODP: Language Model Self-Optimizing Discrete Prompt for Aspect Based Sentiment Analysis

**DOI:** 10.3390/e27121195

**Published:** 2025-11-25

**Authors:** Kun Bu, Yuanchao Liu

**Affiliations:** Faculty of Computing, Harbin Institute of Technology, No. 92 Xidazhi Street, Harbin 150001, China; kunbo@insun.hit.edu.cn

**Keywords:** discrete prompt, aspect based sentiment analysis, reinforcement learning, entropy

## Abstract

Discrete prompts are the main method for interacting with Large Language Models (LLMs) due to their interpretability and cross-model compatibility. However, optimizing them for fine-grained tasks such as Aspect-Based Sentiment Analysis (ABSA) remains challenging, particularly due to error propagation from fixed prediction orders. This problem comes from two issues: errors that cascade in the sequence and the need for intensive human involvement in the prompt design. To solve these problems, we present LM-SODP, a Reinforcement Learning (RL) framework that automatically finds a better discrete prompt and decides a better order to make predictions for ABSA. Our method is based on a distilled GPT-2. It improves how the model uses task-specific information and reduces uncertainty by optimizing the prompts. This reduces the output entropy. LM-SODP also independently finds a better execution sequence for the subtasks in ABSA. Experiments on public datasets show that our method leads to stable improvements under different conditions. By using the optimized prompts, LM-SODP can effectively guide LMs with limited computational resources. It also maintains good performance across different domains and opens new avenues for automated prompt token generation.

## 1. Introduction

The broad adoption of LLMs has improved work efficiency across different tasks. As an effective method to guide LLMs, prompt learning mainly includes two categories: discrete and soft prompts [1]. Although soft prompts generally perform better, they require gradient access and lack cross-model generalizability compared to the discrete prompts. Additionally, many LLMs are only accessible through API (Application Programming Interface), making soft prompts inapplicable. Due to these limitations, most users prefer discrete prompts for LLM interaction. Research shows that LLMs are highly sensitive to prompt instructions, where minor changes can lead to significantly different outputs [1,2,3,4,5]. This has attracted much attention to discrete prompt optimization. However, existing optimization methods rely heavily on human expertise, often failing to align with LLMs’ internal processing patterns. This leads to inefficient information use and poor performance [2,6,7,8].

**Figure 1:**
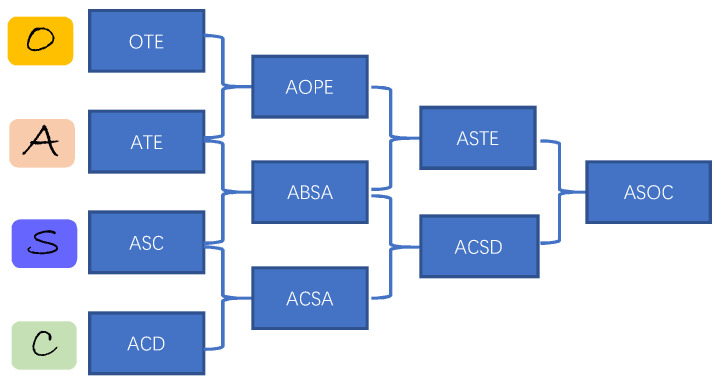
The relationship between ABSA subtasks.

Chain-of-Thought (CoT) represents an advanced prompt learning method that introduces structured, multi-step reasoning processes. By building upon basic prompt instructions, CoT significantly improves the model performance on complex tasks [9]. ABSA is a classic natural language processing task. For example, in the online review “The noodles at this restaurant are delicious, but the beef is average and the pizza is quite unappetizing,” customers express different sentiments toward different aspects (e.g., noodles, beef, and pizza). As shown in Figure 1, ABSA has evolved from a simple three-class (positive, negative, and neutral) sentiment classification into a complex framework consisting of four core subtasks: Aspect Term Extraction (ATE), Opinion Term Extraction (OTE), Aspect Category Detection (ACD), and Aspect Sentiment Classification (ASC) [10,11,12]. Different combinations of these subtasks form new composite tasks, such as Aspect–Opinion Pair Extraction (AOPE), Aspect Category Sentiment Analysis (ACSA), Aspect Sentiment Triplet Extraction (ASTE), Aspect-Category-Sentiment Detection (ACSD), and Aspect Sentiment Opinion Category (ASOC). It should be noted that some tasks may be referred to by multiple names in the literature. Here, we focus primarily on clarifying the relationships between these tasks.

Existing studies indicate that for complex tasks such as ABSA, the prediction order can significantly affect model performance [9]. The inherent logical dependencies among subtasks make ABSA a structurally constrained problem. Existing methods typically use a fixed sequence for predicting meta-tasks, which overlooks the mutual information between related subtasks, such as the natural association between aspects and opinions. This static structure restricts the model’s ability to process information adaptively, leading to information loss and the accumulation of errors across steps, thereby increasing the overall entropy of the meta-task prediction process [13,14]. 

To address these issues, we propose Language Model Self-Optimizing Discrete Prompt (LM-SODP), a method inspired by prior work [2,9,15,16,17]. LM-SODP explores a variety of prompt combinations from different perspectives, simulating the diverse reasoning strategies humans use to solve problems. This approach enhances the model’s robustness and accuracy in prediction. Furthermore, LM-SODP incorporates a multi-order prediction module that optimizes the execution sequence of meta-tasks, effectively reducing conditional entropy and thereby improving the model’s overall information efficiency.

We define the prediction order as the arrangement sequence of sentiment elements (e.g., aspect, category, opinion, and sentiment).

The main contributions of this work can be summarized as the following:

- We introduce LM-SODP, a RL framework for multi chain prompt-based ABSA, achieving better performance than strong baselines such as MvP (Multi-view Prompting) [17] on four tasks with ten datasets.
- LM-SODP demonstrates that lightweight LMs can effectively guide larger LMs, highlighting the potential of prompt learning for broader applications.
- LM-SODP discovers distinctive linguistic patterns within LMs through optimized prompt instructions, which often diverge from human intuition.

The structure of this work is organized as follows: The related work for ABSA, prompt optimization, and RL are introduced in Section 2. We provided a detailed introduction to LM-SODP in Section 3. The results, discussion, and limitations are shown in Section 4. The conclusion is reported in Section 5.

## 2. Related Work

### 2.1. Aspect Based Sentiment Analysis Architectures

SARA addresses the ABSA quadruple extraction task in multi-turn dialogues, improving the model’s capability to capture long-distance dependencies through a span-aware memory block and multi-view mask mechanism [10]. A similar architectural design can also be found in [18]. The integration of Graph Convolutional Networks (GCNs) into ABSA has been widely studied. For instance, Wang et al. proposed a transformer-based model enhanced with GCNs to handle complex textual noise [11]. Similarly, Wang et al. [12] combines GCNs with an autoencoder and capsule-style contrastive learning to better capture subtle semantic and sentiment relationships. Further developments incorporate GCNs with external knowledge and prompt learning [19]. For example, ACM-GT-DAKG (Adaptive Contextual Memory Graph Transformer Domain-Adaptive Knowledge Graph) tackles key challenges—including contextual variability, implicit sentiment reasoning, and limited domain adaptability—through dynamically constructed knowledge graphs and a context-aware, multi-level sentiment modeling framework [20]. Given the close relationships among ABSA sub-tasks, multi-task learning has been effectively used to improve model performance by learning these tasks [15,21]. This strategy has also been combined with GCNs to enhance model capacity [22]. Other recent approaches include the use of diffusion models with syntactic dependency parsing to boost performance [23], and generative models to improve cross-domain adaptability and element extraction capability [24]. Finally, growing evidence confirms the importance of knowledge in ABSA, and the rich prior knowledge embedded in LLMs offers a solid foundation.

### 2.2. Prompt Instruction Optimization

Prompt learning integrates task instructions to guide models in accessing relevant internal knowledge [3,25]. Further developing this approach, Liang et al. bridge discrete tokens with continuous representations, combining the benefits of both optimization strategies to enhance performance while preserving prompt interpretability [16]. Similarly, Chen et al. automate the search for optimal discrete prompts and convert them into continuous forms to improve pre-trained language models (PLMs) [26]. In visual language models (VLMs), discrete prompts demonstrate advantages comparable to those in text-only PLMs. Their strong interpretability and resistance to overfitting have been validated not only in textual tasks but also in cross-modal vision-language applications. This has motivated substantial research on discrete prompt optimization in the vision-language domain [7,8,27]. These studies reflect how discrete prompt optimization has progressed from manual trial-and-error to data-driven automation. This transition has been accompanied by improvements in efficiency through gradient-based optimization and ensemble learning.

### 2.3. Reinforcement Learning in NLP

Based on the Markov Decision Process (MDP) [28], RL provides solutions for various fields through strategies the use of reward functions and other strategies. Landmark systems like AlphaGo [29] have demonstrated its strong potential. However, applying RL to text generation remains challenging. The large discrete action space formed by tokens, combined with sparse reward signals, makes training difficult. Although methods such as deep Q-learning [30] and actor-critic frameworks have been developed, they still struggle to address these fundamental issues. To mitigate reward sparsity, a range of algorithms—including REINFORCE [31], actor-critic [32], hierarchical RL, and inverse RL [33]—have been proposed. Nevertheless, these methods often fall short in handling the rapidly expanding space of discrete tokens effectively. A significant advance was made by Guo et al. [34], who reformulated text generation under the soft Q-learning framework. By establishing a direct link between soft Q-values and model logits, their approach enables the generation model to express the optimal policy naturally, without introducing extra parameters. Furthermore, they adapted path consistency learning to alleviate reward sparsity through a combination of single-step and multi-step objectives. This method effectively overcomes the traditional limitations of inefficiency and instability in the RL-based text generation. The summary of existing literature and mathematical entropy proofs are provided in Appendix B, with further clarification on the role of entropy.

### 2.4. Existing Research Gaps

Prompt learning faces two primary challenges. First, soft prompts must be trained on specific open-source PLMs and demonstrate poor transferability across different models. Meanwhile, existing discrete prompt optimization methods still rely heavily on manual design. Although these methods enhance task performance by enriching the semantic expression of instructions, they often introduce redundant tokens. This not only fails to guarantee improved effectiveness but also incurs unnecessary computational costs. Second, CoT serves as an efficient prompting scheme that guides models through step-by-step reasoning. However, its performance is highly dependent on the reasoning order. Existing CoT methods for ABSA typically adopt a fixed prediction order, overlooking the intrinsic correlations among sentiment elements and leading to error propagation. MvP [17] effectively addresses this issue through its element order-based prompting mechanism. However, MvP relies solely on combinations of element markers (e.g., [A], [C], [O], and [S]) to determine the generation order, without employing explicit instructional prompts for guidance. As a result, its effectiveness remains limited and requires further enhancement.

## 3. Methodology

### 3.1. Discrete Prompt Generation

Building on the success of RL in optimizing discrete prompts for sentence-level tasks [2], we extend this paradigm to the more complex and structured domain of ABSA. Our approach leverages the soft Q-learning framework [34] for effective prompt generation.

For ease of reading, we provide a detailed explanation of the variable meanings in Table 1, we have provided a detailed explanation of the meanings represented by each variable. Discrete prompts are represented by $P=[p_{1},p_{2},p_{3},\ldots ,p_{t}]$. After connecting *P* with the input *x*, the output y(P,x) is obtained by frozen PLM. We define an indicator *R* to evaluate *y*. During the training process, we explore the discrete prompt space by sampling from the policy model. We use soft Q-learning [34] to generate each token individually. For policy network π and task-specific MLP θ (Multi Layer Perceptron), our goal is to maximize the reward signal *R*:

(1) $$\theta^{*}=\arg\max_{\theta }E_{P∼\pi_{\theta }}\left[R(y(P,x))\right].$$

Based on contextual information, we use MLP to obtain the probability distribution of the next token. The final MLP θ is determined through gradient optimization. RL guides the model to learn relevant knowledge by introducing a reward function. Therefore, we introduce normalization to mitigate in the reward function caused by different data. Among the four ABSA meta-tasks, ASC and ACD are text classification tasks, while OTE and ATE are sequence tagging tasks. We design reward functions separately.

For ASC and ACD, we aim to assign *x* the corresponding category label *l* from label set *L*. We design the reward function R(x,l):

(2) $$R\left(x,l\right)=\lambda_{1}^{1-\mathrm{Correct}}\lambda_{2}^{\mathrm{Correct}}\cdot {\mathrm{Gap}}_{p}\left(l\right)$$

where ${\mathrm{Gap}}_{p}\left(l\right)=P_{p}\left(l\right)-\max_{l^{\prime }\neq l}P_{p}\left(l^{\prime }\right)$ indicates the difference between the probability of the target label and the maximum probability of the non-target label, when the classification is correct (Correct=1). The core function of R(x,l) is to encourage high-confidence correct predictions by amplifying the probability gap between correct and incorrect classes.

To provide a clear learning signal, we design a reward function that amplifies both rewards for correct predictions and penalties for incorrect ones. $\lambda_{1}$ and $\lambda_{2}$ are set to 180 and 200.

To address varying classification difficulties across input samples (e.g., larger reward fluctuations in complex sentences), we introduce input-specific z-score normalization:

(3) $$\mathrm{z-score}\left(p,x\right)=\frac{R_{x}\left(p\right)-{\mathrm{mean}}_{p^{\prime }\in P\left(x\right)}R_{x}\left(p^{\prime }\right)}{{\mathrm{stdev}}_{p^{\prime }\in P\left(x\right)}R_{x}\left(p^{\prime }\right)}.$$

For aspect and opinion term extraction (ATE and OTE), we address three key challenges that go beyond standard sequence tagging. We define the sequence labeling task for aspect and opinion term extraction as follows:

- Input sentence: $x=[x_{1},x_{2},\ldots ,x_{n}]$
- Aspect label sequence: $y^{a}=\left[y_{1}^{a},y_{2}^{a},\ldots ,y_{n}^{a}\right]$ where $y_{i}^{a}\in \left\{B,\;I,\;O,\;E,\;S\right\}$
- Opinion label sequence: $y^{o}=\left[y_{1}^{o},y_{2}^{o},\ldots ,y_{n}^{o}\right]$ where $y_{i}^{o}\in \left\{B,\;I,\;O,\;E,\;S\right\}$
- Gold-standard labels: $y^{a*}=\left[y_{1}^{a*},y_{2}^{a*},\ldots ,y_{n}^{a*}\right]$ and $y^{o*}=\left[y_{1}^{o*},y_{2}^{o*},\ldots ,y_{n}^{o*}\right]$

Where B (Begin), I (Inside), O (Outside), S (Single), and E (End).

The optimization of ATE and OTE is motivated by several critical limitations of conventional approaches: First, the inherent cross-label dependency—where each aspect should correspond to at least one opinion, cannot be effectively captured by standard tagging methods. Second, traditional BIO tagging schemes prove inadequate for real-world data, which often contain non-continuous segments. Finally, aspect–opinion pairs must maintain sentiment consistency, a semantic constraint largely overlooked by most sequence labeling models. We reformulate the task as a structured labeling problem. For an input sequence $x=[x_{1},x_{2},\ldots ,x_{n}]$, we predict structured labels $y^{a}\in {\left\{B,I,O,E,S\right\}}^{n}$ for aspects and $y^{o}\in {\left\{B,I,O,E,S\right\}}^{n}$ for opinions. The optimization objective is defined as the following:

(4) $$\max_{z}\left[\lambda R_{\mathrm{ATE}}\left(y^{a}\right)+\left(1-\lambda \right)R_{\mathrm{OTE}}\left(y^{o}\right)\right]$$

where λ∈[0,1] serves as a balancing weight between the rewards for aspect extraction and opinion extraction.

To address the challenge of context-dependent sentiment polarity, our sentiment classifier employs a triplet input formulation: [CLS] complete sentence [SEP] aspect term [SEP] opinion term [SEP]. This input construction enables the classifier to perform sentiment judgement based on specific aspect–opinion–context relationships, rather than relying on isolated word-level sentiment.

Therefore, we designed a three-level reward function:

(5) $$R\left(x\right)=\alpha R_{\mathrm{token}}+\beta R_{\mathrm{pair}}+\gamma R_{\mathrm{sentiment}}$$

where α, β, and γ are weighting coefficients that balance the three reward components. More details can be found in Appendix A.

The optimization objective in Equation (4) aims to balance ATE and OTE through the weighting parameter λ. To implement this objective, we design a unified reward function R(x) in Equation (5) that simultaneously optimizes both tasks.

Specifically, rather than computing separate $R_{\mathrm{ATE}}$ and $R_{\mathrm{OTE}}$, we employ a multi-component reward where:

- $R_{\mathrm{token}}$ incorporates token-level accuracy for both ATE and OTE.
- $R_{\mathrm{pair}}$ evaluates the quality of aspect–opinion pair extraction.
- $R_{\mathrm{sentiment}}$ ensures semantic consistency between aspects and opinions.

The parameter λ in Equation (4) controls the relative emphasis on ATE versus OTE during gradient updates, while the coefficients α, β, and γ in Equation (5) balance the contribution of different reward components to the overall optimization.

Each level of Equation (5) corresponds to a reduction in entropy for a specific dimension. Token level reward guidance models determine whether each character belongs to an aspect, reducing the ambiguity of single token predictions. $R_{\mathrm{token}}$ guided model determines whether each character belongs to an aspect, reducing the ambiguity of single token prediction. $R_{\mathrm{pair}}$ enhances correct pairing, reduces uncertainty in predicting opinions once aspects are identified, and improves the certainty of inter label associations. $R_{\mathrm{sentiment}}$ reduces the joint entropy of emotional polarity judgment, ensuring semantic information consistency and avoiding high entropy interference caused by contradictory labeling.

### 3.2. Multi-Order Prediction

Through discrete prompt optimization, we obtain discrete meta-task instructions. This section introduces multi-perspective reasoning chains. For ease of reading, we provide a detailed explanation of the variable meanings in Table 2. We take the quadruple task as an example. For each sentence *x*, we aim to predict T={(a,c,o,s)}. The corresponding mapping of *T* is $e_{a},e_{c},e_{o},e_{s}$. By arranging and combining the elements, we obtained the predicted sequence $p_{i}$ and target sequence $y_{p_{i}}$:

(6) $$p_{i}\in P\left(e\right)$$

(7) $$y_{p_{i}}=\mathrm{concat}\left(\left[m_{1}\right]e_{m_{1}},\left[m_{2}\right]e_{m_{2}},\ldots ,\left[m_{k}\right]e_{m_{k}}\right)$$

where $\left\{m_{1},m_{2},\ldots ,m_{k}\right\}$ is the element order defined by $p_{i}$ (e.g., $p_{i}=\left[O,A,C,S\right]$ corresponding to $\left[O\right]e_{o}\left[A\right]e_{a}\left[C\right]e_{c}\left[S\right]e_{s}$).

If multiple tuples exist, they are connected using the special symbol [SEP]:

(8) $$y_{p_{i}}=y_{p_{i}}^{\left(1\right)}⊕\left[SEP\right]⊕y_{p_{i}}^{\left(2\right)}$$

where ⊕ represents string concatenation. When the prediction order is [O,A,C,S], we obtain the inference chain $x_{p_{i}}=x⊕\mathrm{prompt}\left(p_{i}\right)$.

We generate all possible prediction orders by combining different arrangements of elements *n* included in the target task P=n!. Using the pre-trained model T5-BASE, we score each permutation $p_{i}$ in P and combinations of sentiment elements, select the top *m* high-scoring sequences, and fine-tune a Seq2Seq model using these sequences.

Specifically, for each arrangement *p*, we calculate its average conditional generation score on the training dataset *D*:

(9) $$D:S_{p_{i}}=\frac{1}{\left|D\right|}\sum_{(x,T)\in D}logp\left(y_{p_{i}}|x\right)$$

where $p\left(y_{p_{i}}|x\right)=\prod_{t=1}^{|y_{p_{i}}|}p\left(y_{t}|x,y_{<t}\right)$. $S_{p_{i}}$ is negatively correlated with predicted entropy.

We then select the top *m* permutations for training. Although *m* is a manually defined hyperparameter, we recommend setting it to an odd number due to the majority voting mechanism. We optimize the Seq2Seq model using negative log-likelihood loss $L_{NLL}$:

(10) $$L_{NLL}=-E_{(x,y)∼D}\sum_{t=1}^{T}\log p_{\theta }\left(y_{t}|x,y_{<t}\right)$$

where *T* represents the target sequence length, and $y_{<t}$ denotes the t−1 tokens that have been generated. During the decoding process, the candidate token set is dynamically restricted The generation path is constrained by the state transition matrix M:

(11) $$M(y_{t+1}\in ValidTokens).$$

This ensures that the output conforms to the target pattern. For the set of tuples generated by *m* permutations $\left\{T_{p_{1}}^{\prime },T_{p_{2}}^{\prime },\ldots ,T_{p_{m}}^{\prime }\right\}$. The final prediction is the voting result $T^{\prime }$:

(12) $$T^{\prime }=\left\{t|t\in \overset{m}{\underset{i=1}{⋃}}T_{p_{i}}^{\prime }\;\mathrm{and}\;\sum_{i=1}^{m}1_{T_{p_{i}}^{\prime }}\left(t\right)\geq \frac{m}{2}\right\}$$

where $1_{T_{p_{i}}^{\prime }}\left(t\right)$ is indicator function, if *t* in $T_{p_{i}}^{\prime }$, t=1, otherwise, t=0.

For different task T and dataset D, we have $x_{\mathrm{multi-task}}$:

(13) $$x_{\mathrm{multi-task}}=\mathrm{TaskName}\left(T\right)⊕\mathrm{DataSetName}\left(D\right)⊕x⊕\mathrm{prompt}\left(p_{i}\right).$$

**Figure 2:**
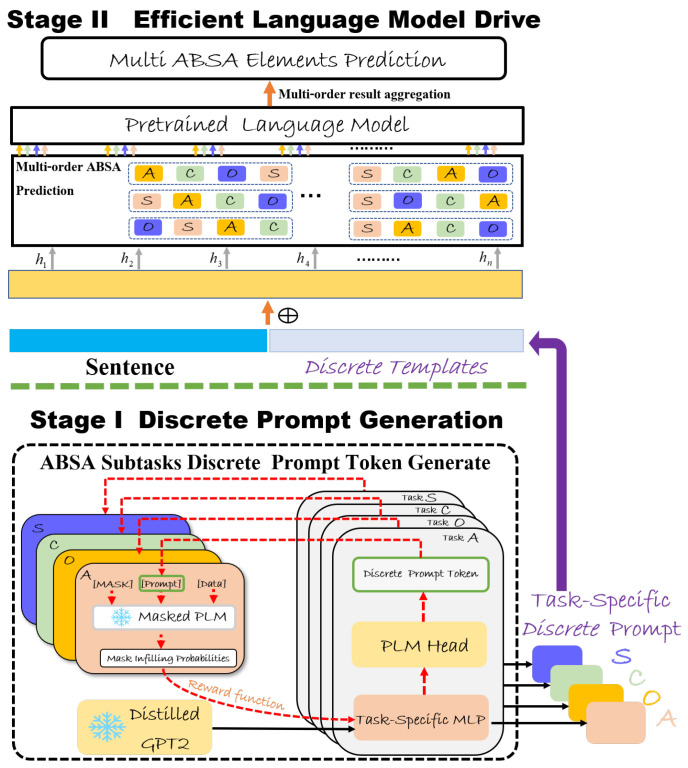
The framework of LM-SODP. The A, C, O, S are the ABSA subtasks. We use different colors to distinguish them. After the discrete prompt instruction is generated, the second stage of emotion information extraction is carried out in the direction indicated by the arrow. The symbol ⊕ is a concatenation of input and prompt instructions. The green square is used to clearly distinguish the first stage from the second stage.

Multi-task learning combines various sentiment tuple prediction tasks using element order prompts within a single model. We prepend task and dataset names to inputs for context, and exclude samples overlapping with the test set to ensure fairness. All training data is mixed and split in a 9:1 ratio for training and validation. By sharing element prediction capabilities, knowledge transfers from simpler to complex tasks. The model adapts to different tuple structures through element arrangement. During training, LM-SODP learns to generate tuples following different orders. During inference, the model generates results using multiple pre-selected orders, and voting aggregation helps reduce errors from any single order. As shown in Figure 2, LM-SODP employs optimized prompts and multiple prediction sequences. Instead of using the single best order, LM-SODP utilizes top m orders (e.g., 15 for quadruples, 5 for triples). This approach selects low-entropy, high-certainty reasoning paths, and the voting mechanism effectively performs an entropy-weighted aggregation, reducing the joint prediction entropy.

We illustrate this process in the example below:

***Input:*** This bread is fantastic! Optimized$Prompt_{order:[A][O][S][C],[A][S][O][C]\ldots }\ldots^{m}$
***Aggregated output:*** [A] bread[O] fantastic[C] food[S] positive

We provided some prompt instruction examples in Table 3 and the pseudocode of LM-SODP in the Appendix B, along with the relevant hyper-parameter settings.

In order to further clarify the relationship between LM-SODP and entropy, we conducted relevant theoretical derivations. As mentioned before, *x* denotes the input sentence and $y_{p_{i}}=\left(a,c,o,s\right)$ represent the target sentiment tuple. As shown in Equation (14), the conditional entropy $H(y_{p_{i}}|x)$ measures the uncertainty in predicting $y_{p_{i}}$ given *x*:

(14) $$H\left(y_{p_{i}}|x\right)=-E_{(x,y)∼D}\left[\log P\left(y|x\right)\right]$$

where D is the data distribution and E denotes the expectation.

For each element order permutation $p_{i}$, LM-SODP computes the average conditional probability, as shown in Equation (15):

(15) $$S_{p_{i}}=E_{D}\left[P\left(y_{p_{i}}|x_{p_{i}}\right)\right]$$

where $x_{p_{i}}=\left[x,p_{i}\right]$ is the prompted input. LM-SODP selects the top-*m* orders with highest $S_{p_{i}}$ values.

The path conditional entropy for order $p_{i}$ is shown in Equation (16):

(16) $$H\left(y_{p_{i}}|x,p_{i}\right)=-E\left[\log P\left(y_{p_{i}}|x,p_{i}\right)\right].$$

Equation (17) shows the monotonicity of the logarithm function and Jensen’s inequality:

(17) $$\max S_{p_{i}}\Leftrightarrow \min-\log S_{p_{i}}\Leftrightarrow \min H\left(y_{p_{i}}|x,p_{i}\right).$$

Thus, selecting orders with high $S_{p_{i}}$ directly minimizes the conditional entropy for each generation path. LM-SODP aggregates predictions through majority voting. This further reduces the uncertainty of predictions. This demonstrates that LM-SODP systematically reduces prediction uncertainty by combining low-entropy generation paths with multi-order aggregation, effectively minimizing the overall conditional entropy $H(y_{p_{i}}|X)$.

## 4. Experiments and Discussion

### 4.1. Datasets and Metrics

As shown in Table 4, we report the dataset composition with key task relationships: ASQP extends ACOS by requiring implicit aspect prediction. ACSD is equivalent to Target Aspect Sentiment Detection (TASD), both detecting sentiment for given aspect categories. ***R*** means restaurant and ***L*** means laptop from SemEval. Some datasets are released based on original dataset. The availability of all related datasets is listed in Table 5 [35,36,37,38,39,40,41]. We use the same data segmentation method as in previous research.

For all tasks, a predicted sentiment tuple is considered correct only if all its elements exactly match the corresponding elements in the gold (ground truth) tuple. If a sentence contains multiple gold tuples, the prediction must include all and only the correct tuples. Each predicted tuple is compared individually against the gold tuples. A predicted tuple is counted as correct only if it has an exact match in the gold set. The overall performance is evaluated using the F1 score.

### 4.2. Baselines

ChatGPT: A generative AI developed by OpenAI. Table A1 and Table A2 list relevant prompt instructions. We use the gpt-3.5-turbo and gpt-4.

Extract–Classify [37]: A novel ACOS quadruple extraction task is proposed to tackle the neglect of implicit aspects and opinions in ABSA, with two datasets constructed.

GAS [42]: A generative framework addresses ABSA’s discriminative method flaws via text generation, using two modeling paradigms and normalization to fit multiple subtasks.

Paraphrase [13]: Authors propose ASQP for quad prediction, using PARAPHRASE paradigm (paraphrase generation) to fit multiple ABSA tasks and build related datasets.

UIE [43]: A unified text-to-structure framework for universal IE, enabling universal task modeling, adaptive structure generation, and cross-source general ability learning.

Seq2Path [44]: Seq2Path addresses ABSA’s Seq2Seq flaws, generating sentiment tuples as tree paths via beam search, constrained decoding and data augmentation.

DLO [14]: A method improves ASQP via template-order data augmentation, selecting proper orders by pre-trained model entropy and using special markers for joint training.

LEGO-ABSA [45]: A prompt-based unified generative framework for ABSA, enabling multi-task training and task transfer by assembling element prompts.

MvP [17]: MvP addresses fixed-order flaws in ABSA tuple prediction via multi-view prompting, generating tuples in varied orders and selecting via voting.

### 4.3. Experiment Results

As reported in Table 6 and Table 7, LM-SODP demonstrates the consistent and significant improvements over strong baselines like MvP, which validates the effectiveness of jointly optimizing prompts and prediction orders. This demonstrates that our method more effectively aligns with the inherent logic of ABSA, mitigating the error propagation that plagues fixed-order approaches. LM-SODP’s performance highlights the advantage of an integrated generation process in avoiding error accumulation.

As shown in Table 8 and Table 9, our experiments evaluate performance under low-resource, cross-task, and cross-domain settings.

Results confirm LM-SODP’s effectiveness in low-resource settings, accurately generating sentiment tuples with just 1% training data. The method demonstrates more stable performance across multiple runs compared to baselines, showing its ability to preserve essential task information through optimized prompts. While ASTE and TASD are inherently simpler than ASQP and ACOS, LM-SODP maintains robust cross-task transfer and cross-domain capability without performance degradation.

**Figure 3:**
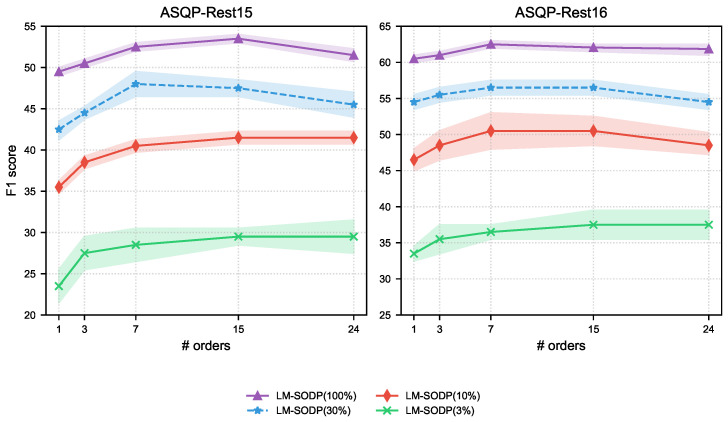
We tested the LM-SODP’s ASQP performance with different prediction order quantities (X-axis) under different training data ratios. All $F_{1}$ scores (Y-axis) are the average of five different random seed experiments. The shaded area is a visual representation of the statistical fluctuation range of experimental results, reflecting the stability of the model.

As shown in Figure 3, the evaluation of LM-SODP on the ASQP task reveals several findings. Performance improves with increasing prediction orders but declines when exceeding 15 orders, suggesting that excessively large orders may introduce noise. The benefits of multiple prediction orders are more substantial in low-resource settings compared to using full training data. However, model stability requires further improvement when training data is limited, as evidenced by fluctuations across multiple runs with different random seeds. Dataset characteristics significantly influence results: Rest15 shows the most pronounced fluctuations at 3% training data due to its smaller size and skewed distribution, while Rest16 exhibits maximum variance at 10% data, reflecting its larger scale and better balance. These results indicate that in low-resource settings, the magnitude of performance fluctuation is closely related to dataset quality.

### 4.4. Ablation Study and Discussion

As shown in Table 10, we conduct ablation studies to analyze the impact of key components in LM-SODP. Our evaluation focuses on two representative tasks, ASTE and ASQP, examining three main aspects: the discrete prompt generation module (replaced with manual prompts), the multi-sequence prediction module (with random, heuristic, and rank variants), and different verbalizers (replacing “terrible/great/neutral” with “negative/positive/neutral” for sentiment tasks).

Across different data scales, we observe several key findings. First, with equivalent training data, LM-SODP’s optimized prompts achieve competitive performance using fewer tokens compared to manual prompts. The prompt generation module demonstrates the most significant impact, confirming that automatically learned prompts guide models more effectively than manually crafted instructions. The fact that automatically optimized prompts—containing seemingly nonsensical token combinations—significantly outperform carefully designed manual prompts indicates that they tap into the model’s internal linguistic patterns rather than human-interpretable semantics.

Our findings demonstrate that label word selection significantly impacts model performance. Concrete, emotionally charged terms like “terrible/great” consistently outperform abstract alternatives like “negative/positive” across experimental settings. This advantage stems from several factors: abstract terms’ polysemy can obscure task intent, verbalizers reshape the language model’s output distribution, and model predictions inherently reflect pre-training co-occurrence patterns. Consequently, verbalizers with strong contextual associations in the pre-training corpus achieve better performance.

LM-SODP shows substantially reduced sensitivity to verbalizer variations compared to manual prompts. Similar patterns emerged in ASQP. This indicates that our automatically optimized prompts can adaptively compensate for suboptimal verbalizer choices.

Our ablation study on prediction order compared three strategies: random element orders, rank-based selection using training-set scores, and heuristic ordering ([A]→[O]→[C]→[S]). Results consistently demonstrate the effectiveness of our multi-order prediction approach over any single fixed-order strategy.

From an information-theoretic perspective, optimized prompts fundamentally differ from manual ones. Manual prompts often misalign with language models’ internal processing, leading to higher prediction uncertainty and greater sensitivity to verbalizer changes. In contrast, LM-SODP’s RL-optimized prompts embed robust task representations that maintain stable performance even with suboptimal verbalizers. The multi-order prediction mechanism enhances robustness through aggregating diverse reasoning paths.

### 4.5. Case Study

**Figure 4:**
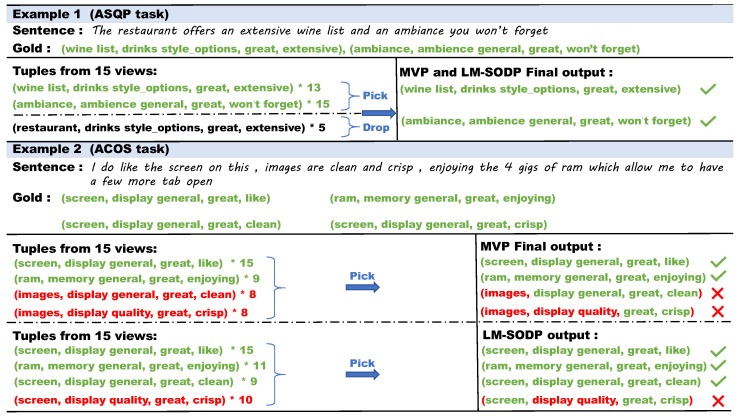
The case study of LM-SODP. The sign ✓ denotes that the model made the correct prediction, while × represents that the model made the wrong prediction. The detailed prediction results are reported in different colored fonts. Green represents correct prediction, while red represents incorrect prediction.

As an important foundation of LM-SODP, MvP points out an important research direction. To highlight the advantages and disadvantages of LM-SODP, we used the same case study as MvP [17]. As shown in Figure 4, experimental results demonstrate that in the ASQP task, both MvP and LM-SODP accurately predict sentiment information at the semantic level, even in cases involving subject confusion. In the ACOS task, MvP produces 15 predictions for the tuple (screen, display general, great, like) and 9 predictions for (ram, memory general, great, enjoying), both exceeding half of the 15-review threshold. Since all elements in these quadruples—aspect term, aspect category, sentiment polarity, and opinion term—exactly match the data label, they are considered correct final outputs. This indicates that MvP can effectively extract sentiment information through rationally designed prediction orders. We believe that the performance gain of RL-based prompt learning stems from its ability to automatically explore token combinations guided by task-specific rewards. The resulting discrete prompts align more effectively with the pre-trained model’s internal linguistic patterns, thereby more efficiently activating its prior knowledge for sentiment information extraction than human-crafted prompts. 

However, for other evaluations targeting the aspect “screen”, incorrect predictions persist. The primary reason lies in the confusion between semantically similar subjects/categories and the inherent challenges of the dataset. Specifically, the Laptop ACOS dataset contains 121 aspect categories with relatively low inter-category semantic distinction, making its prediction more difficult than that of the Restaurant ACOS dataset. The term “images” is strongly associated with “screen” at the semantic level, leading to confusion. By optimizing the prediction order and incorporating higher-quality task-specific prompt instructions, LM-SODP alleviates such semantic mistakes and accurately identifies the aspect “screen”. LM-SODP demonstrates limitations in fine-grained category discrimination, as evidenced by confusion between semantically similar aspects like “*display general*” and “*display quality*”. This underscores the need for enhanced semantic sensitivity in aspect category classification.

### 4.6. Limitations and Future Work

Although LM-SODP has achieved performance improvements over previous research, it still possesses some limitations. First, we acknowledge that the performance gains of LM-SODP come at the cost of increased computational resources. Both training and inference overhead increase linearly with the number of prediction orders *m*. Second, LM-SODP obtains the final model predictions through voting, assigning equal weight to each prediction order, which in rare cases leads to high-quality prediction orders being mistakenly filtered out. Furthermore, in the discrete prompt generation module, we employ soft Q-learning, which is an interchangeable component; however, we did not investigate the performance of other reinforcement learning algorithms. Finally, the decision-making process of LM-SODP is a black box. While we know it synthesizes multiple prediction orders, the reasons why a specific order is selected or discarded remain unclear. When errors occur, it is difficult to trace which order caused the error.

In future work, we will explore more efficient aggregation strategies; test the impact of different reinforcement learning algorithms on ABSA performance; investigate how to balance the number of prediction orders *m* with computational efficiency; and enhance the model’s interpretability and decision transparency.

## 5. Conclusions

This paper presents LM-SODP, a reinforcement learning framework that self-optimizes both discrete prompts and prediction orders for ABSA. Experiments on ten public datasets demonstrate that LM-SODP delivers competitive performance across multiple ABSA tasks. This design enhances the model’s ability to leverage the linguistic knowledge within language models while preserving critical information. Experimental results demonstrate that LM-SODP delivers competitive performance across four distinct ABSA tasks on ten public datasets, even with limited training data, while maintaining strong generalization and robustness. The success of LM-SODP underscores the potential of information-theoretic principles in guiding prompt learning. Notably, our findings show that smaller language models can effectively guide larger ones through optimized prompting. Our work suggests that effective prompting primarily activates the inherent capabilities of language models rather than merely conveying human semantics. LM-SODP not only provides an effective solution for ABSA but also opens new avenues for discrete prompt optimization. The key lies in aligning the model’s information processing with task requirements through appropriate prompt design. It should be noted that the performance of LM-SODP is partly depends on the pre-trained external sentiment classifier used in the reward function, and its compatibility with the target domain.

## Figures and Tables

**Table 1:** Variable definitions for discrete prompt generation.

Variable Name	Symbol	Description
Discrete Prompt	*p*	A sequence of *T* tokens prepended to the input to steer the LM
Prompt Sequence	*P*	Complete prompt sequence containing *t* tokens
Input Text	*x*	The input sentence for the task
Class Label	*l*	The ground-truth label for input *x* from a set *L*
Label Probability	$P_{p}\left(l\right)$	The probability the LM assigns to the true label *l*
Probability Gap	${\mathrm{Gap}}_{p}\left(l\right)$	The difference between the true label’s probability and the highest probability among incorrect labels
Correct Indicator	Correct	An indicator function (1 if prediction is correct, 0 otherwise)
Reward Function	R(x,l)	The reward signal for the RL agent based on classification performance
Reward Weights	$\lambda_{1},\lambda_{2}$	Scaling factors in the reward function to balance correct/incorrect signals.
Policy Network	$\pi_{\theta }$	The parameterized RL policy that generates the prompt tokens
Policy Parameters	θ	The trainable parameters of the policy network (a small MLP)
Vocabulary	V	The set of all possible tokens from which the prompt is built
Aspect Label Sequence	$y^{a}$	BIOES label sequence for aspect terms
Opinion Label Sequence	$y^{o}$	BIOES label sequence for opinion terms
Gold Labels	$y_{i}^{a},y_{i}^{o}$	Gold standard labels for the *i*-th token
Token-level Reward	$R_{\mathrm{token}}$	Reward measuring single token labeling accuracy
Pair-level Reward	$R_{\mathrm{pair}}$	Reward measuring aspect–opinion pairing quality
Sentiment Reward	$R_{\mathrm{sentiment}}$	Reward ensuring sentiment consistency
Reward Weights	α,β,γ	Weight coefficients for three-level rewards
Precision	${\mathrm{PA}}_{\mathrm{prec}}$	Precision for aspect–opinion pairs
Recall	${\mathrm{PA}}_{\mathrm{rec}}$	Recall for aspect–opinion pairs
Training Dataset	*D*	Collection of training examples

**Table 2:** Variable definitions for multi-order prediction.

Variable Name	Symbol	Description
Prediction Order	$p_{i}$	The *i*-th element prediction order permutation
Order Set	P	Set of all possible element permutations
Target Sequence	$y_{p_{i}}$	Target sequence constructed according to order $p_{i}$
Generation Score	$S_{p_{i}}$	Average conditional generation score for permutation $p_{i}$
Predicted Tuple Set	$T_{p_{i}}^{\prime }$	Predicted tuple set generated by order $p_{i}$
Indicator Function	$1_{T_{p_{i}}^{\prime }}\left(t\right)$	Indicator function checking if tuple *t* is in $T_{p_{i}}^{\prime }$
Attention Representation	${\hat{h}}_{i}$	Representation vector after cross-attention
Input Representation	$H_{\mathrm{input}}$	Representation matrix of input text
Aspect Term	*a*	Specific aspect term
Aspect Category	*c*	Predefined category that aspect belongs to
Opinion Term	*o*	Opinion term expressing sentiment
Sentiment Polarity	*s*	Sentiment polarity (positive/negative/neutral).
Element Representation	$e_{a},e_{c},e_{o},e_{s}$	Text representation of corresponding elements (*a*: aspect term, *c*: category, *o*: opinion term, and *s*: polarity)
Sentiment Tuple	{(a,c,o,s)}	Sentiment tuple containing all elements

**Table 3:** The prompt instruction examples. We omitted the preset categories in the ACD task. The meaningless characters in the prompt instructions are automatically generated by the model.

***Task:*** OTE
***Manual Instruction:*** <S>In this task, you are given a sentence. The task is to extract all the specific words or phrases (opinion targets) that express a sentiment (positive, negative, or neutral) in the sentence. It might be ‘null’ for implicit opinion.
***LM-SODP:*** Reviewer Information@@@ [MASK] Features: <S>
***Task:*** ATE
***Manual Instruction:*** <S>In this task, you are given a sentence. The task is to extract all the specific words or phrases (aspect terms) that represent an attribute or feature of an entity being evaluated in the sentence. List only the extracted words/phrases themselves.
***LM-SODP:*** Review tARget@@@ [MASK] Features: <S>
***Task:*** ASC
***Manual Instruction:*** <S>In this task, you are given a sentence and a specific aspect term within that sentence. The task is to classify the sentiment expressed towards that specific aspect term as “positive”, “negative”, or “neutral”.
***LM-SODP:*** <S>Sentiment|||## [ASPECT] @@@Totally => Absolutely[MASK] downright
***Task:*** ACD
**Manual Instruction:** <S>In this task, you are given a sentence and a predefined set of aspect categories. The task is to determine which aspect category (or categories) from the predefined set the sentiment expressed in the sentence belongs to. The sentence may not explicitly mention the category name.
Predefined Aspect Categories: …
***LM-SODP:*** Predefined Aspect Categories: …Category&aspect& <S> » [MASK] reported$$

**Table 4:** Dataset statistics for various tasks. #C is category.

Task	Dataset	#C	Train (/P/Neu/Neg)	Dev (/P/Neu/Neg)	Test (/P/Neu/Neg)
ASQP	R15	13	834 (1005/34/315)	209 (252/14/81)	537 (453/37/305)
	R16	13	1264 (1369/62/558)	316 (341/23/143)	544 (584/40/177)
ACOS	Lap	121	2934 (2583/227/1364)	326 (279/24/137)	816 (716/65/380)
	Rest	13	1530 (1656/95/733)	171 (180/12/69)	583 (668/44/205)
ASTE	L14	–	906 (817/126/517)	219 (169/36/141)	328 (364/63/116)
	R14	–	1266 (1692/166/480)	310 (404/54/119)	492 (773/66/155)
	R15	–	605 (783/25/205)	148 (185/11/53)	322 (317/25/143)
	R16	–	857 (1015/50/329)	210 (252/11/76)	326 (407/29/78)
TASD	R15	13	1120 (1198/53/403)	10 (6/0/7)	582 (454/45/346)
	R16	13	1708 (1657/101/749)	29 (23/1/20)	587 (611/44/204)

**Table 5:** Dataset availability.

Task	Dataset	Availability
ASQP	R15, R16	https://github.com/IsakZhang/ABSA-QUAD (accessed on 3 March 2025)
ACOS	Lap, Rest	https://github.com/NUSTM/ACOS (accessed on 3 March 2025)
ASTE	L14, R14, R15, R16	https://github.com/xuuuluuu/Position-Aware-Tagging-for-ASTE (accessed on 3 March 2025)
TASD	R15, R16	https://github.com/sysulic/TAS-BERT (accessed on 3 March 2025)

**Table 6:** Model performance comparison on ASQP, ACOS, and ACSD datasets. $F_{1}$ scores are reported; All *p*-values were calculated based on paired *t*-tests, with the significance level set at *p* < 0.05. CI denotes the confidence interval of model performance, computed based on five independent experimental runs.

Task Dataset	ASQP		ACOS		ACSD	
	R15	R16	Lap	Rest	R15	R16
ChatGPT-Zero shot (gpt-3.5-turbo)	22.87	–	–	27.11	–	34.08
ChatGPT-Few shot (gpt-3.5-turbo)	34.27	–	–	37.71	–	46.51
ChatGPT-Zero shot (gpt-4)	35.16	–	–	39.64	–	46.71
ChatGPT-Few shot (gpt-4)	45.35	–	–	50.33	–	68.27
Extract-Classify [37]	36.42	43.77	35.80	44.61	–	–
GAS [37]	45.98	56.04	–	–	60.63	68.31
Paraphrase [13]	46.93	57.93	43.51	61.16	63.06	71.97
Seq2Path [44]	–	–	42.97	58.41	63.89	69.23
DLO [14]	48.18	59.79	43.64	59.99	62.95	71.79
LEGO-ABSA [45]	46.10	57.60	–	–	62.30	71.80
SVP (random) [17]	48.32	58.94	43.61	58.16	63.42	71.60
SVP (heuristic) [17]	49.02	59.56	43.83	59.38	61.98	71.57
SVP (rank) [17]	48.39	58.67	43.86	59.57	62.93	71.26
MvP [17]	51.04	60.39	43.92	61.54	64.53	72.76
MvP (multi-task) [17]	52.21	58.94	43.84	60.36	64.74	70.18
LM-SODP	54.53	62.06	46.94	63.27	66.72	73.78
CI	±0.30	±0.56	±0.39	±0.47	±0.62	±0.67
The *p*-value between LM-SODP and MvP	0.021	0.018	0.031	0.019	0.027	0.019

**Table 7:** Model performance comparison on ASTE dataset. $F_{1}$ scores are reported; All *p*-values were calculated based on paired *t*-tests, with the significance level set at *p* < 0.05. CI denotes the confidence interval of model performance, computed based on five independent experimental runs.

Task Dataset	ASTE			
	L14	R14	R15	R16
ChatGPT-Zero shot (gpt-3.5-turbo)	36.05	–	–	–
ChatGPT-Few shot (gpt-3.5-turbo)	38.12	–	–	–
ChatGPT-Zero shot (gpt-4)	48.51	35.14	42.35	46.75
ChatGPT-Few shot (gpt-4)	60.42	40.58	48.35	58.45
GAS [37]	58.19	70.52	60.23	69.05
Paraphrase [13]	61.13	72.03	62.56	71.70
UIE [43]	62.94	72.55	64.41	72.86
Seq2Path [44]	64.82	75.52	65.88	72.87
DLO [14]	61.46	72.39	64.26	73.03
LEGO-ABSA [45]	62.20	73.70	64.40	69.90
SVP (random) [17]	62.36	71.64	62.31	71.59
SVP (heuristic) [17]	62.09	72.61	65.29	73.27
SVP (rank) [17]	63.83	72.71	63.57	71.79
MvP [17]	63.33	74.05	65.89	73.48
MvP (multi-task) [17]	65.30	76.30	69.44	73.10
LM-SODP	66.06	78.16	71.45	75.44
CI	±0.45	±0.49	±0.56	±0.44
The *p*-value between LM-SODP and MvP	0.022	0.033	0.026	0.025

**Table 8:** Low-resource results of LM-SODP. $F_{1}$ scores are reported.

Task	Methods	1%	2%	5%	10%	20%
ASQP (R15)	Paraphrase [13]	5.90	15.73	24.16	31.33	37.47
	DLO [14]	10.03	15.94	29.13	35.89	40.34
	MvP [17]	13.46	22.58	32.44	38.48	41.82
	LM-SODP	14.12	24.18	34.20	40.06	43.77
ACOS (Rest)	Paraphrase [13]	14.85	24.81	38.33	45.32	49.64
	DLO [14]	19.84	29.84	38.47	43.45	46.47
	MvP [17]	23.84	32.57	42.89	47.77	53.54
	LM-SODP	25.62	35.10	44.98	50.01	55.33
TASD (R16)	Paraphrase [13]	26.29	36.70	49.48	55.66	61.79
	DLO [14]	29.66	41.17	50.44	58.27	62.43
	MvP [17]	34.00	41.76	52.58	58.93	64.53
	LM-SODP	34.90	42.13	53.79	60.00	65.75
ASTE (L14)	Paraphrase [13]	16.29	29.20	38.61	45.20	52.88
	DLO [14]	17.07	26.07	38.92	48.85	53.82
	MvP [17]	28.17	34.38	42.89	52.33	54.60
	LM-SODP	30.65	36.28	44.84	55.01	56.37

**Table 9:** Cross-task transfer results of LM-SODP. The sign ^†^ denotes cross-domain. $F_{1}$ scores are reported.

Task	Methods	Transfer Source	1%	2%	5%	10%	20%
ASQP (R15)	DLO (transfer) [14]	ASTE (R15)	26.28	28.72	35.94	39.48	42.92
	MvP (transfer) [17]		28.69	33.93	40.08	43.10	45.09
	LM-SODP (transfer)		30.02	35.11	42.36	44.87	46.54
ACOS (Rest)	DLO (transfer) [14]	ASTE (R16)	31.06	40.55	43.23	45.74	47.98
	MvP (transfer) [17]		39.24	42.72	49.78	52.53	55.28
	LM-SODP (transfer)		41.08	44.07	51.26	53.98	57.09
TASD (R16)	DLO (transfer) [14]	ASQP (R16)	66.25	66.21	64.54	67.99	68.50
	MvP (transfer) [17]		68.49	68.06	68.47	68.98	69.89
	LM-SODP (transfer)		70.11	69.30	70.21	71.16	72.35
ASTE (L14)	DLO (transfer) ^†^ [14]	ASQP (R16)	44.76	48.86	51.22	56.43	56.71
	MvP (transfer) ^†^ [17]		48.43	50.33	54.27	56.34	59.05
	LM-SODP (transfer) ^†^		50.32	52.86	56.73	58.50	61.16

**Table 10:** Ablation study.

Methods	ASTE (L14)			ASQP (R15)		
	1%	10%	100%	1%	10%	100%
LM-SODP	29.35	54.20	66.06	14.58	41.13	54.53
LM-SODP w/o optimized prompt	28.04	51.57	63.12	13.67	38.07	50.76
LM-SODP w/o multi prediction (random)	27.88	51.58	63.21	14.03	38.91	52.11
LM-SODP w/o multi prediction (heuristic)	29.14	54.07	65.94	13.92	39.11	52.37
LM-SODP w/o multi prediction (rank)	28.15	52.61	63.49	13.86	39.08	52.24
LM-SODP with different verbalizers						
- optimized prompt instruction	28.29	53.47	65.63	13.25	40.79	53.84
- manual prompt instruction	26.11	50.15	61.47	11.61	37.16	49.37

## Data Availability

Data will be made available upon request.

## Appendix A

At the token level:

(A1) $$R_{\mathrm{token}}=\frac{1}{2}\left(\frac{1}{n}\sum_{i=1}^{n}I\left(y_{i}^{a}=y_{i}^{a*}\right)+\frac{1}{n}\sum_{i=1}^{n}I\left(y_{i}^{o}=y_{i}^{o*}\right)\right)$$

where I(·) is the indicator function that returns one when the condition is true and zero otherwise.

We balanced the weights of ATE/OTE to prevent the model from being biased towards a single task.

At the pair level:

(A2) $$R_{\mathrm{pair}}=\frac{2\cdot {\mathrm{PA}}_{\mathrm{prec}}\cdot {\mathrm{PA}}_{\mathrm{rec}}}{{\mathrm{PA}}_{\mathrm{prec}}+{\mathrm{PA}}_{\mathrm{rec}}}$$

where ${\mathrm{PA}}_{\mathrm{prec}}$ and ${\mathrm{PA}}_{\mathrm{rec}}$ denote the precision and recall of aspect–opinion pair extraction, respectively.

We addressed the core coupling issue. The sentiment consistency reward:

(A3) $$R_{\mathrm{sentiment}}=-\frac{1}{K}\sum_{k=1}^{K}I\left({\mathrm{Sent}}_{\mathrm{context}}\left(A_{k}\right)\neq {\mathrm{Sent}}_{\mathrm{opinion}}\left(O_{k}\right)\right)$$

where *K* is the number of extracted aspect–opinion pairs, ${\mathrm{Sent}}_{\mathrm{opinion}}\left(O_{k}\right)$ represents the sentiment polarity of opinion term $O_{k}$ in the sentence context, and ${\mathrm{Sent}}_{\mathrm{context}}\left(A_{k}\right)$ represents the inferred sentiment for aspect $A_{k}$ based on the overall sentence context and its associated opinions.

The sentiment consistency reward is designed to ensure alignment between explicitly expressed opinion sentiments and the inferred contextual sentiments of their corresponding aspects. While aspect terms themselves are typically sentiment-neutral, their contextual sentiment is derived from the associated opinions and overall sentence semantics.

$R_{\mathrm{sentiment}}$ incorporated a pre-trained sentiment classifier as a zero-shot verifier. The cross-sequence attention mechanism:

(A4) $${\tilde{h}}_{t}=\mathrm{CrossAttn}\left(\mathrm{PolicyLM}\left(p_{<t}\right),H_{\mathrm{input}}\right)$$

where ${\tilde{h}}_{t}$ dynamically established the association between the prompt token and the input text. The dual-task context fusion is as follows:

(A5) $$\pi_{\theta }\left(p_{t}|\cdot \right)$$

where $\pi_{\theta }\left(p_{t}|\cdot \right)=\mathrm{softmax}\left(W_{o}\left[{\tilde{h}}_{t};\mathrm{AvgPool}\left(H_{\mathrm{input}}\right)\right]\right)$ explicitly injected sentence-level global information to improve long-distance dependencies.

Adaptive normalization:

(A6) $$\mathrm{z-score}\left(p,x\right)=\frac{R_{x}\left(p\right)-\mu_{x}}{\sigma_{x}}$$

where z-score(p,x) was improved to eliminate training bias caused by sentence complexity. Structured prediction enhancement was introduced for non-continuous segments. Nested structure processing is as follows:

(A7) Y←Y∪{A-O:Pos,A-O:Neg}

where Y was used to handle multiple viewpoint conflicts for the same aspect. For non-continuous entity extraction, our method is as follows:

(A8) $$P\left(y^{a}|x\right)\propto \exp\left(\sum_{i=1}^{n}\psi \left(y_{i}^{a},y_{i-1}^{a}\right)+\phi \left(y_{i}^{a},h_{i}\right)\right)$$

(A9) $$P\left(y^{o}|x\right)\propto \exp\left(\sum_{i=1}^{n}\psi \left(y_{i}^{o},y_{i-1}^{o}\right)+\phi \left(y_{i}^{o},h_{i}\right)\right).$$

The CRF (Conditional Random Field) distributions defined in Equations (13) and (14) are proven in [46,47]. This formulation effectively captures label dependencies through transition scores ψ(·) while leveraging contextual representations via emission scores ϕ(·). Compared to alternative distributions such as independent Softmax or structured perceptrons, CRF provides probabilistic guarantees for globally consistent predictions. The choice of CRF balances modeling capacity with computational efficiency in low resource.

## Appendix B

The utility of entropy has been empirically validated in a range of influential RL studies. For instance, Bellemare et al. [48] used entropy as an intrinsic reward, leading to substantial performance gains in complex game environments. Meanwhile, Chen et al. [49] introduced semantic entropy to quantify meaning diversity in multiple generated responses and employed it to modulate policy update magnitudes—demonstrating entropy’s role as a gradient regulator in RL. In the context of LLMs, Cheng et al. [50] explored the relationship between entropy and exploratory reasoning, revealing that high-entropy regions correlate positively with reasoning behaviors. Their study also reflects an evolution in entropy analysis from token-level to step-level granularity. Similarly, Zhang et al. [51] investigated stepwise reasoning in LLMs enhanced by RL, analyzed the limitations of model self-reflection, and studied response-level policy entropy at a fine-grained sample level. To address the sparse reward dependency in the GRPO (Group Relative Policy Optimization) algorithm, EDGE-GRPO was proposed. In parallel, Vanlioglu et al. [52] combined entropy regularization with advantage-weighted learning, effectively balancing policy updates and enabling efficient exploration in high-dimensional state spaces. Collectively, these works underscore a strong and expanding link between RL and entropy.

To convert policy uncertainty into a computable information-theoretic metric, we introduce the Rényi-2 entropy [53] due to it is more suitable for mathematical analysis of RL gradients, as shown in Equation (A1):

(A10) $$H_{2}\left(\pi_{\theta }\left(s\right)\right)=-\log\left(\sum_{a\in A}\pi_{\theta }{\left(a|s\right)}^{2}\right)$$

Where $\pi_{\theta }\left(a|s\right)$ is a strategy with parameter θ, the A is action space.

As the core optimization objects of RL, the policy $\pi_{\theta }$ and its gradient foundation are defined. We explicitly state that the RL objective is gradient ascent to maximize the expected return, as shown in Equations (A2) and (A3):

(A11) $$\pi_{\theta }\left(a|s\right)=\frac{\exp(z_{\theta }\left(a|s\right))}{\sum_{a^{\prime }\in A}\exp\left(z_{\theta }\left(a^{\prime }|s\right)\right)}$$

Where $z_{\theta (a|s)}$ is the action *a* output by LM corresponds to logits.

(A12) $$\frac{\partial \log\pi_{\theta }\left(a|s\right)}{\partial z_{\theta }\left(a^{\prime }|s\right)}=I\left(a^{\prime }=a\right)-\pi_{\theta }\left(a^{\prime }|s\right)$$

Where I() is indicator function, when $\left(a^{\prime }=a\right)$, we obtain I()=1.

Taking the L2 norm of Equation (A3), squaring the resulting norm, expanding the squared term, and performing simplification operations yields Equation (A4):

(A13) $${∥\nabla_{z_{\theta }\left(s\right)}\log\pi_{\theta }\left(a_{k}|s\right)∥}^{2}=1-2\pi_{\theta }\left(a_{k}|s\right)+\sum_{a^{\prime }\in A}\pi_{\theta }{\left(a^{\prime }|s\right)}^{2}$$

where $a_{k}$ is the single action.

Taking the expectation of Equation (A4) with respect to $\pi_{\theta }\left(a|s\right)$, we split the summation terms and simplify them to obtain Equation (A5):

(A14) $$E_{a∼\pi_{\theta }\left(\cdot |s\right)}\left[{∥\nabla_{z_{\theta }\left(s\right)}\log\pi_{\theta }\left(a|s\right)∥}^{2}\right]=1-\sum_{a^{\prime }\in A}\pi_{\theta }{\left(a^{\prime }|s\right)}^{2}.$$

By coupling entropy with gradient magnitude, we derive Equation (A6):

(A15) $$E_{a∼\pi_{\theta }\left(\cdot |s\right)}\left[{∥\nabla_{z_{\theta }\left(s\right)}\log\pi_{\theta }\left(a|s\right)∥}^{2}\right]=1-\exp\left(-H_{2}\left(\pi_{\theta }\left(s\right)\right)\right).$$

In summary, the entropy of independent tokens can be expressed as Equation (A7):

(A16) $$h_{j}=-\sum_{v\in V}p\left(v|w_{<j}\right)\log p\left(v|w_{<j}\right)$$

Where V is a vocabulary, $(p\left(v|w_{<j}\right)$ is the probability of generating the $j_{th}$ token.

The mean of token entropy can be expressed as Equation (A8):

(A17) $$H_{t}=\frac{1}{m_{t}}\sum_{j=1}^{m_{t}}h_{j}$$

Where $m_{t}$ is the number of tokens in step *t*.

After the above deduction, we can conclude that there is a definite mathematical relationship between RL and entropy. We refer interested readers to Wang et al. [54] for more details.

**Table A1:** Zero-shot prompt for ASQP (R15).

Prompt
According to the following sentiment elements definition:
- The ‘aspect term’ refers to a specific feature, attribute, or aspect of a product or service that a user may express an opinion about, the aspect term might be ‘null’ for implicit aspect. - The ‘opinion term’ refers to the sentiment or attitude expressed by a user towards a particular aspect or feature of a product or service, the aspect term might be ‘null’ for implicit opinion. - The ‘aspect category’ refers to the category that aspect belongs to, and the available categories includes: ‘location general’, ‘food prices’, ‘food quality‘, ‘food general’, ‘ambience general’, ‘service general’, ‘restaurant prices’, ‘drinks prices’, ‘restaurant miscellaneous’, ‘drinks quality’, ‘drinks style_options’, ‘restaurant general’ and ‘food style_options’. - The ‘sentiment polarity’ refers to the degree of positivity, negativity or neutrality expressed in the opinion towards a particular aspect or feature of a product or service, and the available polarities includes: ‘positive’, ‘negative’ and ‘neutral’.
Recognize all sentiment elements with their corresponding aspect terms, aspect categories, opinion terms and sentiment polarity in the following text with the format of [(‘aspect term’, ‘opinion term’, ‘aspect category’, ‘sentiment polarity’), …]:

**Table A2:** Few-shot prompt (ten shots) for ASQP (R15).

Prompt and Examples
According to the following sentiment elements definition:
- The ‘aspect term’ refers to a specific feature, attribute, or aspect of a product or service that a user may express an opinion about, the aspect term might be ‘null’ for implicit aspect. - The ‘opinion term’ refers to the sentiment or attitude expressed by a user towards a particular aspect or feature of a product or service, the aspect term might be ‘null’ for implicit opinion. - The ‘aspect category’ refers to the category that aspect belongs to, and the available categories includes: ‘location general’, ‘food prices’, ‘food quality’, ‘food general’, ‘ambience general’, ‘service general’, ‘restaurant prices’, ‘drinks prices’, ‘restaurant miscellaneous’, ‘drinks quality’, ‘drinks style_options’, ‘restaurant general’ and ‘food style_options’. - The ‘sentiment polarity’ refers to the degree of positivity, negativity or neutrality expressed in the opinion towards a particular aspect or feature of a product or service, and the available polarities includes: ‘positive’, ‘negative’ and ‘neutral’.
Recognize all sentiment elements with their corresponding aspect terms, aspect categories, opinion terms and sentiment polarity in the following text with the format of [(‘aspect term’, ‘opinion term’, ‘aspect category’, ‘sentiment polarity’), …]:
Examples:
Text: never again !
Sentiment Elements: [(‘null’, ‘never’, ‘restaurant general’, ‘bad’)]
Text: the food was mediocre at best but it was the horrible service that made me vow never to go back.
Sentiment Elements: [(‘food’, ‘mediocre’, ‘food quality’, ‘bad’), (‘service’, ‘horrible’, ‘service general’, ‘bad’)]
Text: we had the lobster sandwich and it was fantastic.
Sentiment Elements: [(‘lobster sandwich’, ‘fantastic’, ‘food quality’, ‘great’)]
Text: they have it all – great price, food, and service.
Sentiment Elements: [(‘null’, ‘great’, ‘restaurant prices’, ‘great’), (‘food’, ‘great’, ‘food quality’, ‘great’), (‘service’, ‘great’, ‘service general’, ‘great’)]
Text: they even scoop it out nice (for those on a diet) not too much not to little.
Sentiment Elements: [(‘null’, ‘nice’, ‘food style_options’, ‘great’)]
Text: also it’s great to have dinner in a very romantic and comfortable place, the service it’s just perfect …they’re so friendly that we never want to live the place !
Sentiment Elements: [(‘place’, ‘romantic’, ‘ambience general’, ‘great’), (‘place’, ‘comfortable’, ‘ambience general’, ‘great’), (‘service’, ‘perfect’, ‘service general’, ‘great’)]
Text: my friend from milan and myself were pleasantly surprised when we arrived and everyone spoke italian.
Sentiment Elements: [(‘null’, ‘pleasantly surprised’, ‘restaurant miscellaneous’, ‘great’)]
Text: i had their eggs benedict for brunch, which were the worst in my entire life, i tried removing the hollondaise sauce completely that was how failed it was.
Sentiment Elements: [(‘eggs benedict’, ‘worst’, ‘food quality’, ‘bad’)]
Text: the food is authentic italian – delicious !
Sentiment Elements: [(‘food’, ‘authentic italian’, ‘food quality’, ‘great’), (‘food’, ‘delicious’, ‘food quality’, ‘great’)]
Text: a little pricey but it really hits the spot on a sunday morning !
Sentiment Elements: [(‘null’, ‘pricey’, ‘restaurant prices’, ‘bad’), (‘null’, ‘hits the spot’, ‘restaurant general’, ‘great’)]

**Table A3:** Hyperparameters in LM-SODP stage I discrete prompt generation. The weights are determined through grid search on the development set.

Hyperparameter	Description	Value
Policy Network LM	Frozen PLM for prompt generation	Distilled-GPT2 (82 M parameters)
MLP Hidden Size	Trainable MLP layer inserted into policy LM	2048
Training Steps	Total training iterations	20 K
Batch Size	Number of prompts per batch	16
Learning Rate	Adam optimizer learning rate	$5\times 10^{-5}$
Sampling Method	Strategy for generating prompt candidates	top-256 sampling
Reward Scaling Factor	Multiplier to amplify reward signals	5
Reward Normalization	Stabilization technique	Input-specific z-score
Reward Function $\lambda_{1}$	Weight for incorrect predictions	180
Reward Function $\lambda_{2}$	Weight for correct predictions	200
Reward Function α	Ensure the accuracy of basic annotations	0.4
Reward Function β	Modeling aspect-opinion	0.35
Reward Function γ	Ensure sentiment consistency	0.25
Validation Frequency	Steps between validation evaluations	Every 10 steps

**Algorithm A1** Training strategies for ATE and OTE in LM-SODP stage I
**Require:** Task LM (frozen), Policy LM $\pi_{\theta }$, Training set $D_{\mathrm{train}}$ **Require:** α,β,γ ▷ Three-level reward weights **Ensure:** Optimized prompt $z^{*}$ 1: Initialize θ 2: **for** step =1 to $N_{\mathrm{steps}}$ **do** 3: $\mathrm{prompts\_batch}\leftarrow \mathrm{GeneratePrompts}(\pi_{\theta },“\mathrm{sequence\_labeling}”,T,“\mathrm{top-256}”)$ 4: rewards←∅ 5: **for** each z in prompts_batch **do** 6: $R_{\mathrm{total}}\leftarrow 0$ 7: **for** each $\left(x,y^{a},y^{o}\right)$ in $D_{\mathrm{train}}$ **do** 8: ${\hat{y}}^{a},{\hat{y}}^{o}\leftarrow \mathrm{EnhancedDecode}\left(z,x\right)$ 9: $R\leftarrow \mathrm{ThreeLevelReward}({\hat{y}}^{a},{\hat{y}}^{o},y^{a},y^{o})$ 10: $R_{\mathrm{total}}\leftarrow R_{\mathrm{total}}+R$ 11: **end for** 12: $R_{\mathrm{avg}}\leftarrow R_{\mathrm{total}}/D_{\mathrm{train}}$ 13: $\mathrm{rewards}\leftarrow \mathrm{rewards}\cup \{R_{\mathrm{avg}}\}$ 14: **end for** 15: rewards←ZscoreNormalization(rewards) ▷ Reward stabilization 16: rewards←rewards×5 ▷ Reward scaling 17: $\theta \leftarrow \mathrm{SQL\_Update}(\pi_{\theta },\mathrm{prompts\_batch},\mathrm{rewards})$ ▷ Soft Q-learning update 18: **if** step mod10=0 **then** 19: $\mathrm{val\_perf}\leftarrow \mathrm{EvaluateOnValidationSet}(\pi_{\theta })$ 20: LogPerformance(step,val_perf) 21: **end if** 22: **end for** 23: $z^{*}\leftarrow \mathrm{GreedyDecode}\left(\pi_{\theta },“\mathrm{sequence\_labeling}”,T\right)$ 24: **return** $z^{*}$ 25: **function** EnhancedDecode(z,x) 26: input_with_prompt←concat(z,x) 27: Apply CRF for structured prediction ▷ Handle sequence labeling structure 28: Handle boundary constraints and nested structures ▷ For complex span detection 29: **return** ${\hat{y}}^{a},{\hat{y}}^{o}$ ▷ Predicted aspect and opinion labels 30: **end function** 31: **function** ThreeLevelReward(${\hat{y}}^{a},{\hat{y}}^{o},y^{a},y^{o}$) 32: $R_{\mathrm{token}}\leftarrow \mathrm{TokenLevelAccuracy}\left({\hat{y}}^{a},{\hat{y}}^{o},y^{a},y^{o}\right)$ 33: $R_{\mathrm{pair}}\leftarrow \mathrm{AspectOpinionF}1\left({\hat{y}}^{a},{\hat{y}}^{o},y^{a},y^{o}\right)$ 34: $R_{\mathrm{sentiment}}\leftarrow \mathrm{SentimentConsistency}\left({\hat{y}}^{a},{\hat{y}}^{o},y^{a},y^{o}\right)$ 35: **return** $\alpha R_{\mathrm{token}}+\beta R_{\mathrm{pair}}+\gamma R_{\mathrm{sentiment}}$ 36: **end function**

**Table A4:** Hyperparameters and hardware information in LM-SODP stage II.

Hyperparameters	LM-SODP	LM-SODP (Low Resource)			
		1%, 2%, 3%, 5%	10%, 20%	30%	50%
Epoch	20	100	50	30	20
Batch Size	32	16			
Learning Rate	$1\times 10^{-4}$				
Optimizer	AdamW				
GPU	Nvidia RTX 3090 * 2				
CUDA Version	11.6				
System	ubuntu22.04				
Python Version	3.8				
GPU Memory Used	about 40 GB				
Runing Time	about 6 h				

**Algorithm A2** Training strategies for ASC and ACD in LM-SODP stage I
**Require:** Task LM (frozen), Policy LM $\pi_{\theta }$, Training set $D_{\mathrm{train}}=\left\{\left(x_{i},y_{i}\right)\right\}$, Prompt length *T* **Ensure:** Optimized discrete prompt $z^{*}$ 1: Initialize policy network parameters θ 2: **for** step =1 to $N_{\mathrm{steps}}$ **do** 3: prompts_batch←∅ 4: **for** i=1 to batch_size **do** 5: $z\leftarrow \mathrm{GeneratePrompt}(\pi_{\theta },“\mathrm{classification}”,T,“\mathrm{top-256}”)$ 6: prompts_batch←prompts_batch∪{z} 7: **end for** 8: rewards←∅ 9: **for** each prompt z in prompts_batch **do** 10: reward_sum←0 11: **for** each example (x,c) in $D_{\mathrm{train}}$ **do** 12: input_with_prompt←concat(z,x) 13: $P_{z}\leftarrow \mathrm{Task\_LM}\left(\mathrm{input\_with\_prompt}\right)$ 14: $\mathrm{gap}\leftarrow P_{z}\left(c\right)-\max_{c^{\prime }\neq c}P_{z}\left(c^{\prime }\right)$ 15: correct←[gap>0] 16: $R_{xc}\leftarrow \left(\lambda_{1}^{1-\mathrm{correct}}\cdot \lambda_{2}^{\mathrm{correct}}\right)\cdot \mathrm{gap}$ ▷$\lambda_{1}$ = 180, $\lambda_{2}$ = 200 17: $\mathrm{reward\_sum}\leftarrow \mathrm{reward\_sum}+R_{xc}$ 18: **end for** 19: $R_{\mathrm{avg}}\leftarrow \mathrm{reward\_sum}/D_{\mathrm{train}}$ 20: $\mathrm{rewards}\leftarrow \mathrm{rewards}\cup \{R_{\mathrm{avg}}\}$ 21: **end for** 22: rewards←ZscoreNormalization(rewards) 23: rewards←rewards×5 ▷ Reward scaling 24: $\theta \leftarrow \mathrm{SQL\_Update}(\pi_{\theta },\mathrm{prompts\_batch},\mathrm{rewards})$ 25: **if** step mod10=0 **then** 26: $\mathrm{val\_acc}\leftarrow \mathrm{EvaluateOnValidationSet}(\pi_{\theta })$ 27: LogPerformance(step,val_acc) 28: **end if** 29: **end for** 30: $z^{*}\leftarrow \mathrm{GreedyDecode}\left(\pi_{\theta },“\mathrm{classification}”,T\right)$ 31: **return** $z^{*}$

**Algorithm A3** Multi-order result aggregation strategies in LM-SODP stage II
**Require:** Training dataset D={(x,T)} where optimized specific task prompt instructions T={(a,c,o,s)}; Pre-trained LM; Number of orders *m* **Ensure:** Trained model *M* for inference 1: * * **Training Phase:** 2: *Step 1: Element Order Selection* 3: P← all possible element permutations 4: **for** each permutation $p_{i}\in P$ **do** 5: $S_{p_{i}}\leftarrow \frac{\sum_{D}p\left(y_{p_{i}}|x\right)}{D}$ 6: **end for** 7: $P_{\mathrm{selected}}\leftarrow \mathrm{top-}m\;\mathrm{permutations}\;\mathrm{ranked}\;\mathrm{by}\;S_{p_{i}}$ 8: *Step 2: Multi-order Training* 9: **for** each (x,T)∈D **do** 10: **for** each $p_{i}\in P_{\mathrm{selected}}$ **do** 11: $x_{p_{i}}\leftarrow x+T_{p_{i}}$ 12: $y_{p_{i}}\leftarrow$ construct target sequence with markers [A],[C],[O],[S] 13: Fine-tune LM using $L_{NLL}=-E\log p\left(y|x\right)$ 14: **end for** 15: **end for** 16: M← trained model 17: * * **Inference Phase:** 18: **procedure** LM-SODP Inference($x,M,P_{\mathrm{selected}}$) 19: Predictions←[] 20: **for** each $p_{i}\in P_{\mathrm{selected}}$ **do** 21: $x_{p_{i}}\leftarrow x+p_{i}$ 22: Generate $T_{p_{i}}^{\prime }$ with ConstrainedDecoding 23: $\mathrm{Predictions}.\mathrm{append}(T_{p_{i}}^{\prime })$ 24: **end for** 25: *Aggregate results via majority voting:* 26: $T_{\mathrm{LM-SODP}}^{\prime }\leftarrow \left\{t|\sum_{i=1}^{m}1_{T_{p_{i}}^{\prime }}\left(t\right)\geq m/2\right\}$ 27: **return** $T_{\mathrm{LM-SODP}}^{\prime }$ 28: **end procedure** 29: * * **Constrained Decoding:** 30: **function** ConstrainedDecoding(current_token) 31: **if** current_token ∈{[A],[O]} **then** 32: **return** {tokensfrominput,[SSEP]} 33: **else if** current_token =[S] **then** 34: **return** {“positive”,“negative”,“neutral”,[SSEP]} 35: **else if** current_token =[C] **then** 36: **return** {allcategories,[SSEP]} 37: **else** 38: **return** full vocabulary 39: **end if** 40: **end function**
